# Longitudinal effects of gender minority stressors on substance use and related risk and protective factors among gender minority adolescents

**DOI:** 10.1371/journal.pone.0250500

**Published:** 2021-06-02

**Authors:** Sabra L. Katz-Wise, Vishnudas Sarda, S. Bryn Austin, Sion Kim Harris

**Affiliations:** 1 Division of Adolescent/Young Adult Medicine, Boston Children’s Hospital, Boston, MA, United States of America; 2 Department of Pediatrics, Harvard Medical School, Boston, MA, United States of America; 3 Department of Social and Behavioral Sciences, Harvard T. H. Chan School of Public Health, Boston, MA, United States of America; Universidad de Granada, SPAIN

## Abstract

**Purpose:**

Gender minority (GM) adolescents, who have a different gender identity than their sex assigned at birth, may use substances as a coping strategy in response to GM-related stressors. This study examined longitudinal effects of gender minority stressors on substance use in GM adolescents, and related risk factors (internalized transphobia, depressive symptoms, anxious symptoms) and protective factors (resilience, gender-related pride, family functioning, social support, gender-related community connectedness).

**Methods:**

Participants were 30 GM adolescents, ages 13–17 years, from the U.S. community-based longitudinal Trans Teen and Family Narratives Project. Participants completed an online survey every 6 months across 2 years (5 waves; data collected 2015–2019).

**Results:**

Exposure to gender minority stressors was associated with higher odds of alcohol use. Across models, internalized transphobia (risk factor), resilience (protective factor), and gender-related pride (protective factor) were the most significant mediators of associations between gender minority stressors and substance use. Family functioning and social support (protective factors) significantly moderated the association between gender minority stressors and alcohol use, such that family functioning and social support were protective for alcohol use at lower levels of gender minority stress, but not at higher levels.

**Conclusion:**

Results suggest that GM adolescents engage in substance use as a coping strategy in response to gender minority stressors. A number of hypothesized risk and protective factors mediated or moderated these associations. Future interventions with GM adolescents should focus efforts on addressing internalized transphobia as a risk factor and strengthening resilience, gender-related pride, and family functioning as protective factors for substance use.

## Introduction

Increased substance use is common among adolescents, as youth experiment with strategies to cope with life stressors. For gender minority (GM) adolescents, who have a different gender identity than their sex assigned at birth, exposure to GM-related stressors, such as victimization, may result in substance use as a coping mechanism [1, 2], which may negatively affect GM adolescents’ health. Compared to cisgender adolescents, GM adolescents report higher rates of risk factors (e.g., emotional distress, bullying victimization) and lower rates of protective factors (e.g., internal assets, family connectedness) [2].

GM adolescents are at higher risk for substance use than cisgender adolescents [2, 3]. Previous research indicated the prevalence of substance use was 2.5–4 times higher for GM youth compared to cisgender youth.^3^ Substance use among GM adolescents may be linked to experiencing GM-related stressors, such as stigma-based prejudice [1, 2, 4]. A recent study of cisgender sexual minority and GM adolescents found that experiencing multiple types of victimization was more common among GM than cisgender sexual minority youth [1].

Minority stress research has indicated GM individuals experience adverse mental health outcomes and use substances to cope with experiences of prejudice [3, 5–8]. The U.S. National Transgender Discrimination Survey reported that 28% of adult transgender men used substances to cope with GM-related stigma in healthcare [5]. Compared to cisgender adolescents, GM adolescents report higher rates of emotional distress [2], which may increase the likelihood of using substances to cope with minority stress [9]. A recent study found higher prevalence of substance use among GM youth compared to cisgender youth was partially explained by victimization and depressive symptoms [3]. Protective factors may buffer adverse effects of minority stress on GM adolescents’ mental health. A study conducted by our team found better family functioning was associated with less self-harm and depressive/anxious symptoms, and greater self-esteem and resiliency among GM adolescents [10], indicating family support is an important protective factor for GM youth. One of the few studies to examine protective factors related to substance use among GM adolescents found that family connectedness and social support may reduce the likelihood that GM adolescents will use substances to cope with minority stressors [9, 10]. Other GM-specific protective factors, such as gender-related pride and gender-related community connectedness, may also reduce substance use among GM adolescents, though to our knowledge, this has not yet been studied. Previous research examining associations between minority stressors and substance use among GM adolescents was cross-sectional; longitudinal methods are needed to establish patterns over time.

Study aims were to examine longitudinal effects of gender minority stressors on substance use among GM adolescents and to identify related risk and protective factors for intervention efforts. Notably, some risk and protective factors are relevant for all adolescents (depressive/anxious symptoms, resilience, family functioning, social support) and some risk and protective factors are relevant specifically for GM adolescents (internalized transphobia, gender-related pride, gender-related community connectedness). Thus, this study aimed to examine how risk and protective factors relevant to all adolescents specifically operate in GM adolescents related to their substance use, as well as examining some GM-specific risk and protective factors. We hypothesized that: H1) greater exposure to gender minority stressors would be longitudinally associated with greater substance use over time among GM adolescents, H2) risk factors (internalized transphobia, depressive/anxious symptoms) would mediate longitudinal associations between gender minority stressors and substance use, and H3) protective factors (mediators: resilience, gender-related pride; moderators: family functioning, social support, gender-related community connectedness) would either mediate or moderate (depending on the specific factor) longitudinal associations between gender minority stressors and substance use among GM adolescents.

## Methods

### Participants

Participants were 33 GM adolescents, ages 13–17 years (at Wave 1), from the longitudinal Trans Teen and Family Narratives (TTFN) Project. Participants were included in this analysis if they completed the relevant measures at Wave 1 and at least one additional wave (analytic N = 30). Participants identified as trans feminine (n = 11), trans masculine (n = 15), and nonbinary assigned female (n = 4). Most participants identified their race/ethnicity as White (73%) or Hispanic/Latinx (13%). See Table 1 for other sample characteristics.

**Table 1:** Sample characteristics at Wave 1 (N = 30).

Measure	All Participants
Age, M (SD), range: 13–17 years	15.1 (1.1)
Gender identity, n (%)	
Trans feminine	11 (36.7)
Trans masculine	15 (50.0)
Non-binary	4 (13.3)
Sex assigned at birth, n (%)	
Female	18 (60.0)
Male	12 (40.0)
Race/ethnicity, n (%)	
White	22 (73.3)
Hispanic or Latinx	4 (13.3)
Asian, Native Hawaiian or other Pacific Islander	2 (6.7)
American Indian or Alaska Native	1 (3.3)
Mixed race/ethnicity	1 (3.3)
Sexual orientation identity, n (%)	
Completely straight/heterosexual	6 (20.0)
Mostly straight/heterosexual	2 (6.7)
Bisexual	5 (16.7)
Mostly lesbian/gay	5 (16.7)
Completely lesbian/gay	4 (13.3)
Queer	7 (23.3)
Pansexual	13 (43.3)
Questioning	2 (6.7)
Substance use	
Tobacco	
Ever used, n (%)	4 (13.3%)
Age at first use, M (SD), range: 13–14 years	13.7 (0.6)
Alcohol	
Ever used, n (%)	8 (26.7%)
Age at first use, M (SD), range: 12–16 years	13.4 (1.3)
Marijuana	
Ever used, n (%)	7 (23.3%)
Age at first use, M (SD), range: 13–16 years	14.4 (1.0)

### Study design

Participants completed an online survey at five waves, every six months across two years (December 2015 to March 2019). Surveys were completed following a qualitative interview at the participants’ home, at the researchers’ offices, or via video conference for participants who lived beyond a 2.5-hour driving radius from researcher offices. GM adolescents participated in the study with 1–2 caregivers and a sibling. Survey data from GM adolescents only were analyzed for the current study. Prior to completing the interview and survey, each participant gave written informed assent or consent. Surveys were administered by researchers who were LGBTQ-identified or allies. Following study participation, each participant received a $20 gift card and a list of resources tailored for GM adolescents and their families. A comprehensive safety plan was used to follow-up with participants who reported intention to harm themselves or others in the interview or psychological distress on the survey (score of ≥11 on the CES-D) and connect them to mental health screening and resources. This study was approved by the Boston Children’s Hospital Institutional Review Board.

### Measures

#### Predictor: Gender minority stressors

*Gender-minority stressor composite*. Three subscales from the Gender Minority Stress and Resilience Measure (GMSR) [11]: gender-related rejection, non-affirmation of gender identity, and gender-related victimization, were combined into one composite scale. Gender-related rejection (5 items, sample item: “I have been rejected or distanced from friends because of my gender identity or expression”) and non-affirmation of gender identity (6 items, sample item: “I have to repeatedly explain my gender identity to people or correct the pronouns people use”) were both assessed at Waves 1–5. Wave 1 response options were modified to fit the age of the current sample: 0 (Never); 1 (Yes, before age 13); 1 (Yes, after age 13); 1 (Yes, in the past year). Gender-related victimization (6 items, sample item: “I have been threatened with physical harm because of my gender identity or expression”) was assessed at Waves 2–5. Only 2 of the 6 items, verbal harassment and threats of physical harm, were endorsed by participants in this sample, and therefore included in the composite score. Response options for all three subscales at Waves 2–5 assessed experiences from the past 6 months: 0 (Never), 1 (Yes, but not in the past 6 months), 1 (Yes, in the past 6 months). An overall composite scale score was calculated for each wave by summing responses across all items (range: 0–11); higher scores indicated greater exposure to gender-minority stressors. Cronbach’s alpha was 0.73 for the composite scale.

#### Outcomes: Substance use

Tobacco, alcohol, and marijuana use were assessed at Waves 1–5 with items from the Youth Risk Behavior Surveillance System Survey (YRBS) [12]. Participants ages 13–14 years completed items from the middle school survey; participants ages 15–17 years completed items from the high school survey. When younger participants became age 15–17 years in later waves, they completed items from the high school survey.

Each substance was assessed with a yes/no question from the YRBS [12] (“Have you ever tried cigarette smoking, even one or two puffs?”; “Have you ever had a drink of alcohol, other than a few sips?”; “Have you ever used marijuana?”). For alcohol use assessed in Waves 2–5, this question was changed to “In the past 6 months, have you ever had a drink of alcohol, other than a few sips?” (yes/no). If participants responded yes to any substance at any wave, they answered 2 additional items assessing age of first use (both age groups) and past 30-day use (both age groups for tobacco; age 15–17 only for alcohol and marijuana).

#### Risk mediators

*Internalized transphobia*. Assessed at Waves 1–5 with an 8-item subscale from the GMSR [11]. Sample item: “When I think of my gender identity or expression, I feel depressed.” Response options were on a 5-point Likert scale: 0 (strongly disagree) to 4 (strongly agree). We calculated a subscale score for each wave by summing item responses (range: 0–24); higher scores indicated greater internalized transphobia. Cronbach’s alpha was 0.90.

*Depressive symptoms*. Assessed for the past week with the 10-item Center for Epidemiologic Studies Depression Scale (CES-D)–Short Form [13]. Sample item: “I was bothered by things that usually don’t bother me.” Response options: 0 (rarely or none of the time–less than 1 day), 1 (some or a little of the time– 1–2 days), 2 (occasionally or a moderate amount of time– 3–4 days), 3 (most or all of the time– 5–7 days). Items worded in the opposite valence were reverse-coded and a scale score was created by summing item responses (range: 0–30); higher scores indicated greater depressive symptoms. Cronbach’s alpha was 0.88.

*Anxious symptoms*. Assessed with the 6-item Spence Children’s Anxiety Scale (SCAS) [14, 15]. Sample item: “I worry about things.” Response options: 1 (never), 2 (sometimes), 3 (often), 4 (always). A scale score was created by summing item responses (range: 6–24); higher scores indicated greater anxious symptoms. Cronbach’s alpha was 0.87.

#### Protective mediators

*Resilience*. Assessed with the 22-item Resilience Scale for Adolescents [16]. Sample item: “I reach my goals if I work hard.” Response options were on a 5-point Likert scale: 1 (totally disagree) to 5 (totally agree). A scale score was calculated by computing a mean of the items (range: 1–5); higher scores indicated greater resiliency. Cronbach’s alpha was 0.91.

*Gender-related pride*. Assessed at Waves 1–5 with an 8-item subscale from the GMSR [11]. Sample item: “My gender identity or expression makes me feel special and unique.” Response options were on a 5-point Likert scale: 0 (strongly disagree) to 4 (strongly agree). A subscale score was calculated for each wave by summing item responses (range: 0–32); higher scores indicate greater gender-related pride. Cronbach’s alpha was 0.83.

#### Moderators

*Family functioning*. Assessed with two subscales from the Family Adaptability and Cohesion Evaluation Scales (FACES IV) [17]. Family communication subscale (10 items): Sample item: “Family members are very good listeners.” Response options were on a 5-point Likert scale: 1 (strongly disagree) to 5 (strongly agree). Family satisfaction subscale (10 items): Sample item: “The degree of closeness between family members.” Response options were on a 5-point Likert scale: 1 (very dissatisfied) to 5 (extremely satisfied). The two subscales were combined into one scale. An overall scale score was calculated for each wave by summing responses across items from both subscales (range: 20–100); higher scores indicated better family functioning. Cronbach’s alpha was 0.95 for the combined subscales.

*Social support*. Assessed with the 12-item Multidimensional MSPSS [18]. Sample item: “There is a special person who is around when I am in need.” Response options were on a 7-point Likert scale: 1 (very strongly disagree) to 7 (very strongly agree). Scale scores were calculated by summing item responses (range: 12–84); higher scores indicated more perceived support. Cronbach’s alpha was 0.92.

*Gender-related community connectedness*. Assessed at Waves 1–5 with a 5-item subscale from the GMSR [11]. Sample item: “I feel part of a community of people who share my gender identity.” Response options were on a 5-point Likert scale: 0 (strongly disagree) to 4 (strongly agree). A subscale score was calculated for each wave by summing item responses (range: 0–20); higher scores indicate greater gender-related community connectedness. Cronbach’s alpha was 0.81.

### Statistical analyses

**Fig 1:**
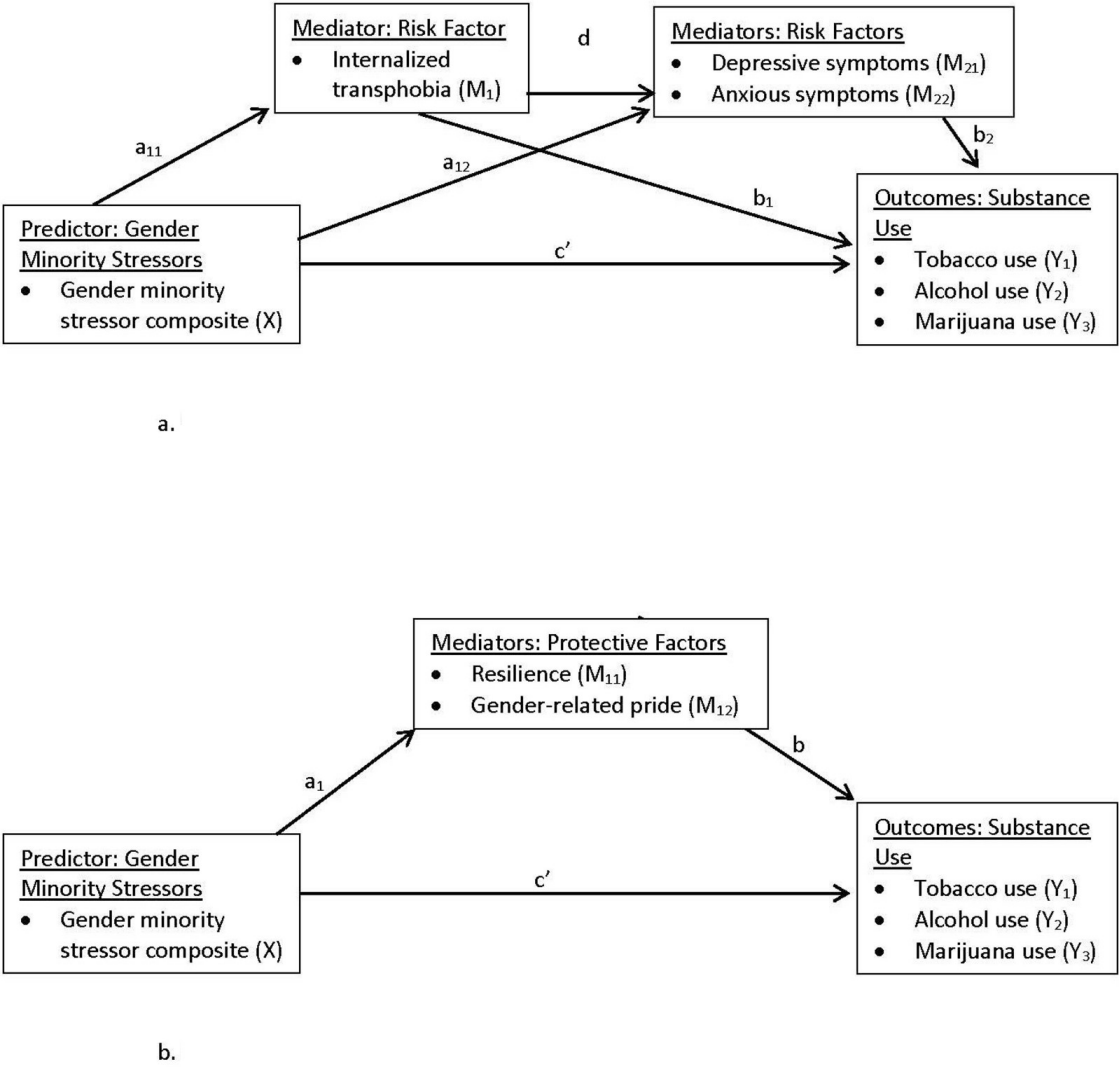
(a) Multiple Mediation Statistical Model with Mediation by Risk Factors of the Longitudinal Effect of Gender Minority Stressors on Substance Use Across Waves 1–5. (b) Mediation Statistical Model with Mediation by Protective Factors of the Longitudinal Effect of Gender Minority Stressors on Substance Use Across Waves 1–5.

We computed univariate statistics (frequency for categorical variables, M/SD for continuous variables) for all predictors, outcomes, and hypothesized mediators/moderators across Waves 1–5 (i.e., across two years). We assessed change over time in gender minority stressors and substance use, using generalized linear mixed effects modeling with data nested within participant, and wave as a continuous predictor. We ran these models accounting for the multiple observations per participant by including a random intercept; we examined wave as a fixed effect. To evaluate potential mediation/moderation of the association between gender minority stressors (fixed predictor) and reported use of each type of substance (outcomes) at the subsequent wave by our hypothesized risk and protective factors, we first examined bivariate associations between gender minority stressors and the hypothesized mediator/moderator variables. Bivariate associations were examined using regression analysis with generalized estimating equations (GEE), with data nested within subjects, to account for intra-subject correlation of data across Waves 1–5. We then used Hayes’ approach to test three mediation and moderation models [19]. Two mediation models tested for mediation of the associations between gender minority stressors and substance use by risk factors (H1) and protective factors (H2). The first mediation model (Fig 1A) tested a risk pathway by which gender minority stressors were associated with greater substance use across two years via sequential mediating internal risk factors (internalized transphobia leading to depressive/anxious symptoms). The second mediation model (Fig 1B) tested a protective pathway by which gender minority stressors were associated with less substance use across two years via mediating internal protective factors (resilience, gender-related pride). Finally, models tested for moderation of associations between gender minority stressors and substance use by social factors (family functioning, social support, gender-related community connectedness) (H3). Models predicting alcohol and marijuana use were adjusted for wave and sex assigned at birth (both treated as fixed effects); models predicting tobacco use were adjusted only for sex assigned at birth due to the low rate of tobacco use across waves. Wave was used as a covariate rather than age because age data were missing and the two variables were highly correlated. Participants were excluded from analyses if they were missing data across Waves 2–5 (n = 3).

## Results

At Wave 1, 17% of participants reported any substance use and 4% reported multiple substance use (Table 2). Regarding specific substances, at Wave 1, 13% of participants had ever used tobacco, 27% had ever used alcohol, and 23% had ever used marijuana (Table 1). Among participants who had ever used substances at Wave 1, the average age of first use was 13.7 years for tobacco, 13.4 years for alcohol, and 14.4 years for marijuana (Table 1). By Wave 5 (two years after Wave 1), 56% of participants reported any substance use and 32% reported multiple substance use (Table 2).

**Table 2:** Descriptive statistics for predictors, outcomes, and hypothesized moderators for Waves 1–5 (N = 30).

	Wave 1	Wave 2	Wave 3	Wave 4	Wave 5	Beta (SE)^1^	QIC
Predictors: Gender minority stressors	n = 30	n = 25	n = 25	n = 25	n = 29		
Gender minority stressor composite, M (SD), range: 0–11	3.8 (2.9)	2.3 (2.3)	3.1 (2.9)	2.6 (2.3)	1.6 (1.6)	-0.42 (0.13)**	138.6
Outcomes: Substance use^2^	n = 29	n = 25	n = 21	n = 20	n = 25		
Recent tobacco use, n (%)	0 (0.0%)	1 (4.0%)	2 (9.6%)	6 (30.0%)	5 (20.0%)	0.71 (0.17)*	80.9
Recent alcohol use, n (%)	3 (10.3%)	4 (16.0%)	6 (28.6%)	9 (45.0%)	10 (40.0%)	0.47 (0.14)*	134.8
Recent marijuana use, n (%)	3 (10.3%)	5 (20.0%)	4 (19.1%)	7 (35.0%)	10 (40.0%)	0.42 (0.13)*	131.7
Any substance use, n (%)	5 (17.2%)	8 (32.0%)	8 (38.1%)	12 (60.0%)	14 (56.0%)	0.46 (0.14)**	154.9
Poly substance use (≥ 2 substances), n (%)	1 (3.5%)	2 (8.0%)	3 (14.3%)	7 (35.0%)	8 (32.0%)	0.63 (0.16)**	104.4
Hypothesized mediators: Risk factors	n = 30	n = 25	n = 25	n = 25	n = 29		
Internalized transphobia, M (SD), range: 0–24	15.8 (5.9)	15.5 (7.3)	17.6 (6.5)	15.9 (6.5)	15.0 (6.2)	-0.15 (0.35)	137.9
Depressive symptoms, M (SD), range: 0–30	11.7 (7.4)	9.4 (6.5)	10.2 (6.9)	10.9 (6.6)	9.3 (6.4)	-0.36 (0.39)	139.7
Anxious symptoms, M (SD), range: 6–24	14.4 (4.7)	13.4 (4.4)	13.4 (4.2)	14.4 (4.4)	13.2 (3.5)	-0.15 (0.22)	138.7
Hypothesized mediators: Protective factors	n = 30	n = 25	n = 25	n = 25	n = 29		
Resilience, M (SD), range: 1–5	3.6 (0.5)	3.7 (0.5)	3.7 (0.6)	3.6 (0.7)	3.7 (0.6)	-0.01 (0.02)	141.0
Gender-related pride, M (SD), range: 0–32	29.3 (5.7)	28.0 (5.3)	29.4 (6.5)	27.6 (6.3)	28.1 (6.0)	-0.28 (0.24)	139.8
Hypothesized moderators	n = 30	n = 25	n = 25	n = 25	n = 29		
Family functioning, M (SD), range: 20–100	71.5 (12.7)	74.6 (12.8)	72.5 (15.6)	71.9 (14.5)	74.3 (13.9)	0.35 (0.56)	140.3
Social support, M (SD), range: 12–84	69.5(11.1)	70.3 (8.8)	68.4(14.2)	69.5(10.1)	69.7 (8.9)	-0.02 (0.51)	139.4
Gender-related community connectedness, M (SD), range: 0–20	19.6 (3.9)	19.0 (3.5)	19.1 (3.6)	19.6 (3.4)	18.9 (3.6)	-0.10 (0.20)	138.8

^1^Models tested change over time (trend) using wave as a continuous predictor in GLM with GEE; coefficients for mediators and moderators tested differences between time points using wave as a categorical predictor in GLM with GEE.

*p < .05

**p < .01.

^2^For all types of substance use, recent use = use in the past 6 months.

### Gender minority stressors and substance use

Results indicated that rates of recent tobacco, alcohol, and marijuana use all significantly increased across two years (Table 2). Higher exposure to gender minority stressors significantly increased the odds of alcohol use in later waves (OR = 1.59, 95% CI = 1.02, 2.49), but not of tobacco or marijuana use; therefore, H1 was partially supported.

### Risk pathway models: Mediation by risk factors

Bivariate coefficients indicated strong positive associations between gender minority stressor exposure and hypothesized risk mediators (Table 3; see Supplemental S1 Table for QIC). In all analyzed models, higher gender minority stressor exposure was associated with higher odds of internalized transphobia, and depressive and anxious symptoms across two years (paths a_11_ and a_12_, Table 4). Internalized transphobia, in turn, was associated with increased odds of depressive and anxious symptoms over time. Internalized transphobia also had a significant independent effect on subsequent substance use risk in all models, except in the tobacco use mediation model with depressive symptoms as the second mediator. Consequently, internalized transphobia was a significant mediator of the effect of gender minority stressor exposure on subsequent substance use across all models, except for the tobacco use model in which depressive symptoms was the second mediator (Table 4); in that model, depressive symptoms was the significant mediator. In all other models, however, depressive/anxious symptoms did not show a significant independent effect on substance use risk. As a result, tests of the indirect effect through both internalized transphobia and depressive/anxious symptoms (i.e., multiple sequential mediators) were largely non-significant. Across models, internalized transphobia was more consistently a significant mediator than either depressive or anxious symptoms. In sum, H2 was partially supported.

**Table 3:** Bivariate associations among gender minority stressors and hypothesized risk and protective factors.

Measures	1	2	3	4	5	6	7	8	9
1. Gender minority stressor composite	1								
2. Internalized transphobia	0.38***	1							
3. Depressive symptoms	0.53***	0.37***	1						
4. Anxious symptoms	0.27**	0.07	0.52***	1					
5. Resilience	-0.37**	-0.45***	-0.65***	-0.40**	1				
6. Gender-related pride	-0.45***	-0.35**	-0.49***	-0.29**	0.34***	1			
7. Family functioning	-0.24*	-0.25*	-0.23**	-0.09	0.14**	0.33**	1		
8. Social support	-0.18*	-0.45**	-0.33***	-0.19*	0.29***	0.19***	0.33***	1	
9. Gender-related community connectedness	-0.03	-0.14*	-0.10	0.08	-0.02	0.13**	0.14*	0.23***	1

*p < .05

**p < .01

***p < .001.

**Table 4:** Risk factor mediation models: Results from serial multiple mediation models^1^ testing risk mediators of the longitudinal effect of gender minority stressors on substance use across Waves 1–5.

	Tobacco Use (Y_1_)			
	Depressive Symptoms (M_21_)		Anxious Symptoms (M_22_)	
Measures	Path	OR (95% CI)	Path	OR (95% CI)
Gender Minority Stressor Composite to Internalized Transphobia	a_11_ (X to M_1_)	**1.58 (1.35, 1.87)**	a_11_ (X to M_1_)	**1.58 (1.35, 1.87)**
Gender Minority Stressor Composite to Depressive/Anxious Symptoms	a_12_ (X to M_21_)	**1.55 (1.32, 1.82)**	a_12_ (X to M_22_)	**1.38 (1.14, 1.68)**
Internalized Transphobia to Tobacco Use	b_1_ (M_1_ to Y_1_)	1.58 (0.76, 3.31)	b_1_ (M_1_ to Y_1_)	**2.08 (1.07, 4.04)**
Depressive/Anxious Symptoms to Tobacco Use	b_2_ (M_21_ to Y_1_)	2.05 (0.89, 4.69)	b_2_ (M_22_ to Y_1_)	0.75 (0.32, 1.78)
Internalized Transphobia to Depressive/Anxious Symptoms	d (M_1_ to M_21_)	**1.41 (1.20, 1.66)**	d (M_1_ to M_22_)	1.07 (0.88, 1.30)
Gender Minority Stressor Composite to Tobacco Use	c’ (X to Y_1_)	1.07 (0.62, 2.11)	c’ (X to Y_1_)	1.57 (0.78, 3.14)
Indirect Effect through Internalized Transphobia	X to M_1_ to Y_1_	1.23 (0.88, 2.20)	X to M_1_ to Y_1_	**1.40 (1.04, 2.72)**
Indirect Effect through Depressive/Anxious Symptoms	X to M_21_ to Y_1_	**1.36 (1.01, 2.53)**	X to M_22_ to Y_1_	0.91 (0.58, 1.21)
Indirect Effect through Internalized Transphobia and Depressive/Anxious Symptoms	X to M_1_ to M_21_ to Y_1_	**1.12 (1.00, 1.40)**	X to M_1_ to M_22_ to Y_1_	0.99 (0.91, 1.04)
	Alcohol Use (Y_2_)			
	Depressive Symptoms (M_21_)		Anxious Symptoms (M_22_)	
Measures	Path	OR (95% CI)	Path	OR (95% CI)
Gender Minority Stressor Composite to Internalized Transphobia	a_11_ (X to M_1_)	**1.58 (1.34, 1.87)**	a_11_ (X to M_1_)	**1.58 (1.34, 1.87)**
Gender Minority Stressor Composite to Depressive/Anxious Symptoms	a_12_ (X to M_21_)	**1.55 (1.32, 1.82)**	a_12_ (X to M_22_)	**1.38 (1.13, 1.67)**
Internalized Transphobia to Alcohol Use	b_1_ (M_1_ to Y_2_)	**2.81 (1.51, 5.26)**	b_1_ (M_1_ to Y_2_)	**2.32 (1.34, 4.00)**
Depressive/Anxious Symptoms to Alcohol Use	b_2_ (M_21_ to Y_2_)	0.60 (0.30, 1.20)	b_2_ (M_22_ to Y_2_)	0.89 (0.49, 1.59)
Internalized Transphobia to Depressive/Anxious Symptoms	d (M_1_ to M_21_)	**1.41 (1.20, 1.66)**	d (M_1_ to M_22_)	1.07 (0.88, 1.30)
Gender Minority Stressor Composite to Alcohol Use	c’ (X to Y_2_)	1.42 (0.78, 2.60)	c’ (X to Y_2_)	1.20 (0.69, 2.08)
Indirect Effect through Internalized Transphobia	X to M_1_ to Y_2_	**1.62 (1.23, 2.86)**	X to M_1_ to Y_2_	**1.48 (1.15, 2.34)**
Indirect Effect through Depressive/Anxious Symptoms	X to M_21_ to Y_2_	0.80 (0.55, 1.05)	X to M_22_ to Y_2_	0.96 (0.73, 1.19)
Indirect Effect through Depressive Symptoms and Depressive/Anxious Symptoms	X to M_1_ to M_21_ to Y_2_	0.92 (0.78, 1.01)	X to M_1_ to M_22_ to Y_2_	1.00 (0.95, 1.03)
	Marijuana Use (Y_3_)			
	Depressive Symptoms (M_21_)		Anxious Symptoms (M_22_)	
Measures	Path	OR (95% CI)	Path	OR (95% CI)
Gender Minority Stressor Composite to Internalized Transphobia	a_11_ (X to M_1_)	**1.58 (1.34, 1.87)**	a_11_ (X to M_1_)	**1.58 (1.34, 1.87)**
Gender Minority Stressor Composite to Depressive/Anxious Symptoms	a_12_ (X to M_21_)	**1.55 (1.32, 1.82)**	a_12_ (X to M_22_)	**1.38 (1.13, 1.67)**
Internalized Transphobia to Marijuana Use	b_1_ (M_1_ to Y_3_)	**1.92 (1.11, 3.33)**	b_1_ (M_1_ to Y_3_)	**2.10 (1.24, 3.56)**
Depressive/Anxious Symptoms to Marijuana Use	b_2_ (M_21_ to Y_3_)	1.00 (0.56, 1.80)	b_2_ (M_22_ to Y_3_)	0.43 (0.22, 0.83)
Internalized Transphobia to Depressive/Anxious Symptoms	d (M_1_ to M_21_)	**1.41 (1.20, 1.66)**	d (M_1_ to M_22_)	1.07 (0.88, 1.30)
Gender Minority Stressor Composite to Marijuana Use	c’ (X to Y_3_)	0.82 (0.47, 1.44)	c’ (X to Y_3_)	1.11 (0.63, 1.97)
Indirect Effect through Internalized Transphobia	X to M_1_ to Y_3_	**1.35 (1.06, 1.88)**	X to M_1_ to Y_3_	**1.40 (1.13, 2.01)**
Indirect Effect through Depressive/Anxious Symptoms	X to M_21_ to Y_3_	1.00 (0.78, 1.34)	X to M_22_ to Y_3_	0.76 (0.49, 0.91)
Indirect Effect through Depressive Symptoms and Depressive/Anxious Symptoms	X to M_1_ to M_21_ to Y_3_	1.00 (0.91, 1.12)	X to M_1_ to M_22_ to Y_3_	0.97 (0.90, 1.05)

^1^Tobacco models controlled for sex assigned at birth; alcohol and marijuana models controlled for both wave and sex assigned at birth. Significant effects are bolded.

### Protective pathway models: Mediation by protective factors

Across models, gender minority stressor exposure was significantly negatively associated with the protective factors of resilience and gender-related pride (Table 5). Increased resilience and gender-related pride, in turn, tended to predict lower odds of subsequent substance use (all adjusted odds ratios below 1), although the effects were only statistically significant for tobacco (resilience and gender-related pride) and marijuana use (resilience only). This protective mediation pathway was significant for tobacco use (through gender-related pride) and for marijuana use (through resilience). In sum, H3 was partially supported.

**Table 5:** Protective factor mediation models: Results from mediation models^1^ testing protective mediators of the longitudinal effect of gender minority stressors on substance use across Waves 1–5.

	Tobacco Use (Y_1_)			
	Resilience (M_11_)		Gender-Related Pride (M_12_)	
Measures	Path	OR (95% CI)	Path	OR (95% CI)
Gender Minority Stressor Composite to Resilience/Gender-Related Pride	a_1_ (X to M_11_)	**0.68 (0.57, 0.81)**	a_1_ (X to M_12_)	**0.75 (0.63, 0.91)**
Resilience/Gender-Related Pride to Tobacco Use	b (M_11_ to Y_1_)	**0.48 (0.23, 1.00)**	b (M_12_ to Y_1_)	**0.40 (0.19, 0.82)**
Gender Minority Stressor Composite to Tobacco Use	c’ (X to Y_1_)	1.53 (0.85, 2.77)	c’ (X to Y_1_)	1.54 (0.85, 2.79)
Indirect Effect through Resilience/Gender-Related Pride	X to M_11_ to Y_1_	1.32 (0.41, 2.34)	X to M_12_ to Y_1_	**1.30 (1.00, 2.27)**
	Alcohol Use (Y_2_)			
	Resilience (M_11_)		Gender-Related Pride (M_12_)	
Measures	Path	OR (95% CI)	Path	OR (95% CI)
Gender Minority Stressor Composite to Resilience/Gender-Related Pride	a_1_ (X to M_11_)	**0.68 (0.57, 0.81)**	a_1_ (X to M_12_)	**0.75 (0.63, 0.90)**
Resilience/Gender-Related Pride to Alcohol Use	b (M_11_ to Y_1_)	0.67 (0.40, 1.11)	b (M_12_ to Y_1_)	0.67 (0.40, 1.12)
Gender Minority Stressor Composite to Alcohol Use	c’ (X to Y_1_)	1.42 (0.89, 2.24)	c’ (X to Y_1_)	1.46 (0.93, 2.29)
Indirect Effect through Resilience/Gender-Related Pride	X to M_11_ to Y_2_	1.17 (0.98, 1.55)	X to M_12_ to Y_2_	1.12 (0.97, 1.40)
	Marijuana Use (Y_3_)			
	Resilience (M_11_)		Gender-Related Pride (M_12_)	
Measures	Path	OR (95% CI)	Path	OR (95% CI)
Gender Minority Stressor Composite to Resilience/Gender-Related Pride	a_1_ (X to M_11_)	**0.68 (0.57, 0.81)**	a_1_ (X to M_12_)	**0.75 (0.63, 0.90)**
Resilience/Gender-Related Pride to Marijuana Use	b (M_11_ to Y_1_)	**0.59 (0.36, 0.96)**	b (M_12_ to Y_1_)	0.71 (0.45, 1.13)
Gender Minority Stressor Composite to Marijuana Use	c’ (X to Y_1_)	0.92 (0.58, 1.47)	c’ (X to Y_1_)	1.01 (0.65, 1.56)
Indirect Effect through Resilience/Gender-Related Pride	X to M_11_ to Y_3_	**1.23 (1.03, 1.58)**	X to M_12_ to Y_3_	1.11 (0.95, 1.32)

^1^Tobacco models controlled for sex assigned at birth; alcohol and marijuana models controlled for both wave and sex assigned at birth. Significant effects are bolded.

### Moderation effects

**Fig 2:**
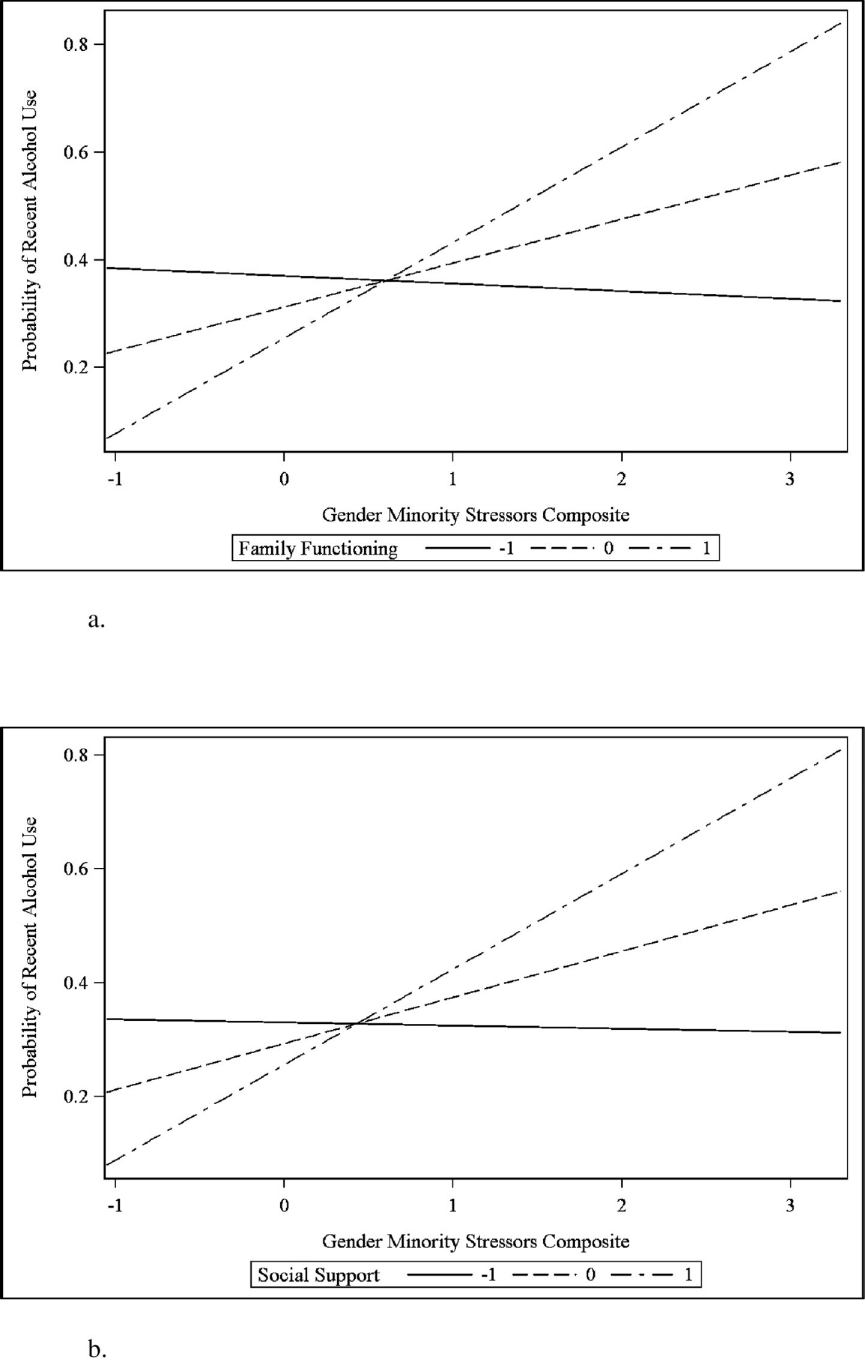
(a) Significant Interaction Between Gender Minority Stressor Composite and Family Functioning Predicting Alcohol Use (-1 = lower family functioning; 1 = higher family functioning). (b) Significant Interaction Between Gender Minority Stressor Composite and Social Support Predicting Alcohol Use (-1 = lower social support, 1 = higher social support).

We found that family functioning and social support both significantly moderated the effect of gender minority stressor exposure on alcohol use across two years (Table 6), with family functioning and social support protective for alcohol use at lower levels of gender minority stress, but not at higher levels of gender minority stress (Fig 2A and 2B). We found no other significant moderation effects. In sum, H3 was partially supported.

**Table 6:** Results from models^1^ testing moderators of the longitudinal effect of gender minority stressors on substance use across Waves 1–5.

Measures	Tobacco Use	Alcohol Use	Marijuana Use
	OR (95% CI)	OR (95% CI)	OR (95% CI)
Family Functioning Model			
Gender Minority Stressor Composite	1.41 (0.72, 2.76)	1.83 (0.96, 3.49)	0.64 (0.34, 1.19)
Family Functioning	0.74 (0.29, 1.95)	0.51 (0.25, 1.04)	**0.30 (0.15, 0.59)**
Gender Minority Stressor Composite x Family Functioning	0.70 (0.28, 1.74)	**2.38 (1.35, 4.21)**	0.94 (0.61, 1.44)
Social Support Model			
Gender Minority Stressor Composite	1.51 (0.70, 3.26)	**1.67 (1.05, 2.66)**	0.91 (0.55, 1.50)
Social Support	0.55 (0.24, 1.27)	0.70 (0.38, 1.29)	0.47 (0.21, 1.02)
Gender Minority Stressor Composite x Social Support	0.83 (0.38, 1.84)	**1.87 (1.17, 3.00)**	0.99 (0.56, 1.74)
Gender-Related Community Connectedness Model			
Gender Minority Stressor Composite	1.83 (0.79, 4.21)	**1.59 (1.01, 2.49)**	1.07 (0.63, 1.79)
Gender-Related Community Connectedness	0.64 (0.35, 1.18)	0.70 (0.37, 1.35)	0.78 (0.42, 1.45)
Gender Minority Stressor Composite x Gender-Related Community Connectedness	1.30 (0.69, 2.43)	1.36 (0.79, 2.33)	0.97 (0.60, 1.57)

^1^Tobacco models controlled for sex assigned at birth; alcohol and marijuana models controlled for both wave and sex assigned at birth. Significant effects are bolded.

## Discussion

This longitudinal study examined prospective associations between exposure to gender minority stressors and substance use across two years among GM adolescents, with nearly 60% of participants reporting recent substance use at any wave by the final time point. At the final time point, 20% of the GM adolescents in the current study reported recent tobacco use, and 40% reported recent alcohol use and marijuana use. For comparison, data from the Youth Risk Behavior Survey collected during 2019 (data for the current study were collected in December 2015 to March 2019) indicated that 37% of high school students reported recent tobacco use, 29% reported recent alcohol use, and 22% reported recent marijuana use [20]. Thus, GM adolescents the in current study reported higher rates of alcohol and marijuana use, but not tobacco use. In the current study, exposure to gender minority stressors was associated with higher odds of alcohol use, but not higher odds of tobacco or marijuana use. These findings support previous minority stress research indicating that GM individuals engage in substance use (namely alcohol) to cope with prejudice [3, 5–8].

In testing hypothesized mediation pathways between gender minority stressor exposure and substance use, we found that internalized transphobia, resilience, and gender-related pride significantly mediated risk or protective pathways between exposure to gender minority stressors and substance use. Notably, two of these three factors are specific to GM: internalized transphobia and gender-related pride. Internalized transphobia is comprised of four separate dimensions: lack of pride in GM identity, desire to pass as a cisgender person, isolation from other GM people, and shame [21]. GM adolescents may need support to reduce internalized transphobia and bolster gender-related pride, ultimately reducing substance use in this population.

Results also indicated family functioning and social support moderated associations between gender minority stressor exposure and alcohol use, such that family functioning and social support were protective for alcohol use at lower levels of gender minority stress, but not at higher levels of gender minority stress. This is consistent with previous research demonstrating the protective role of social support and family connectedness for GM adolescents’ substance use [8]. Interestingly, family functioning and social support were not protective for alcohol use at higher levels of gender minority stress, highlighting the need for more research to identify protective factors. Still, family functioning and social support were protective at lower levels of gender minority stress and family functioning was independently associated with lower odds of marijuana use among GM youth in this sample. Substance use interventions for this population should focus on improving family functioning and increasing social support within and outside of the family.

A number of limitations should be mentioned; the sample was small, and disproportionately White and non-Hispanic and higher socioeconomic status. Participants were also geographically limited to the New England region of the U.S. Findings may not be generalizable to GM groups underrepresented in this sample. The measures used at Wave 1 assessed a different timeframe than the measures used at Waves 2–5, which limits comparability of the data across waves. The sample size was also too small to allow for modeling wave as a multinomial variable; thus, wave was modeled as a linear variable. An additional limitation is that the Hayes approach to modeling mediation/moderation did not allow for specification of longitudinal correlation structure of data, resulting in greater risk for Type 1 error. Despite limitations, the TTFN Project provides a unique opportunity to examine longitudinal effects of gender minority stressor exposure on substance use across two years among GM adolescents, as a first step for future research in this area.

## Conclusion

Findings from this research have implications for intervention efforts to reduce substance use among GM adolescents. Efforts should focus on addressing internalized transphobia as a risk factor and strengthening resilience, gender-related pride, family functioning, and social support as protective factors for substance use. Since GM adolescents appear to be using substances to cope with exposure to gender minority stressors, programs could assist adolescents in identifying adaptive coping strategies in response to such stressors. GM adolescents should also be connected to resources where they can connect with other GM adolescents. Efforts on a macro-level to increase anti-discrimination policies and laws and decrease stigma toward GM individuals may ultimately improve the lives of GM adolescents by reducing exposure to gender minority stressors.

## Supporting information

S1 Table Goodness-of-Fit (QIC) for bivariate associations among gender minority stressors and hypothesized risk and protective factors. (DOCX)
