# Correction: Longitudinal effects of gender minority stressors on substance use and related risk and protective factors among gender minority adolescents

**DOI:** 10.1371/journal.pone.0304725

**Published:** 2024-05-29

**Authors:** Sabra L. Katz-Wise, Vishnudas Sarda, S. Bryn Austin, Sion Kim Harris

There is an error in the article PDF causing the last heading in Table 4 to be incorrect. The heading should be: Marijuana Use. Please see the correct Table 4 here.

**Table 4:** Risk Factor Mediation Models: Results from Serial Multiple Mediation Models1 Testing Risk Mediators of the Longitudinal Effect of Gender Minority Stressors on Substance Use Across Waves 1–5

	**Tobacco Use (Y_1_)**			
	**Depressive Symptoms (M_21_)**		**Anxious Symptoms (M_22_)**	
**Measures**	**Path**	**OR (95% CI)**	**Path**	**OR (95% CI)**
Gender Minority Stressor Composite to Internalized Transphobia	a_11_ (X to M_1_)	**1.58 (1.35, 1.87)**	a_11_ (X to M_1_)	**1.58 (1.35, 1.87)**
Gender Minority Stressor Composite to Depressive/Anxious Symptoms	a_12_ (X to M_21_)	**1.55 (1.32, 1.82)**	a_12_ (X to M_22_)	**1.38 (1.14, 1.68)**
Internalized Transphobia to Tobacco Use	b_1_ (M_1_ to Y_1_)	1.58 (0.76, 3.31)	b_1_ (M_1_ to Y_1_)	**2.08 (1.07, 4.04)**
Depressive/Anxious Symptoms to Tobacco Use	b_2_ (M_21_ to Y_1_)	2.05 (0.89, 4.69)	b_2_ (M_22_ to Y_1_)	0.75 (0.32, 1.78)
Internalized Transphobia to Depressive/Anxious Symptoms	d (M_1_ to M_21_)	**1.41 (1.20, 1.66)**	d (M_1_ to M_22_)	1.07 (0.88, 1.30)
Gender Minority Stressor Composite to Tobacco Use	c’ (X to Y_1_)	1.07 (0.62, 2.11)	c’ (X to Y_1_)	1.57 (0.78, 3.14)
Indirect Effect through Internalized Transphobia	X to M_1_ to Y_1_	1.23 (0.88, 2.20)	X to M_1_ to Y_1_	**1.40 (1.04, 2.72)**
Indirect Effect through Depressive/Anxious Symptoms	X to M_21_ to Y_1_	**1.36 (1.01, 2.53)**	X to M_22_ to Y_1_	0.91 (0.58, 1.21)
Indirect Effect through Internalized Transphobia and Depressive/Anxious Symptoms	X to M_1_ to M_21_ to Y_1_	**1.12 (1.00, 1.40)**	X to M_1_ to M_22_ to Y_1_	0.99 (0.91, 1.04)
	**Alcohol Use (Y**_**2**_ **)**			
	**Depressive Symptoms (M**_**21**_ **)**		**Anxious Symptoms (M**_**22**_ **)**	
**Measures**	**Path**	**OR (95% CI)**	**Path**	**OR (95% CI)**
Gender Minority Stressor Composite to Internalized Transphobia	a_11_ (X to M_1_)	**1.58 (1.34, 1.87)**	a_11_ (X to M_1_)	**1.58 (1.34, 1.87)**
Gender Minority Stressor Composite to Depressive/Anxious Symptoms	a_12_ (X to M_21_)	**1.55 (1.32, 1.82)**	a_12_ (X to M_22_)	**1.38 (1.13, 1.67)**
Internalized Transphobia to Alcohol Use	b_1_ (M_1_ to Y_2_)	**2.81 (1.51, 5.26)**	b_1_ (M_1_ to Y_2_)	**2.32 (1.34, 4.00)**
Depressive/Anxious Symptoms to Alcohol Use	b_2_ (M_21_ to Y_2_)	0.60 (0.30, 1.20)	b_2_ (M_22_ to Y_2_)	0.89 (0.49, 1.59)
Internalized Transphobia to Depressive/Anxious Symptoms	d (M_1_ to M_21_)	**1.41 (1.20, 1.66)**	d (M_1_ to M_22_)	1.07 (0.88, 1.30)
Gender Minority Stressor Composite to Alcohol Use	c’ (X to Y_2_)	1.42 (0.78, 2.60)	c’ (X to Y_2_)	1.20 (0.69, 2.08)
Indirect Effect through Internalized Transphobia	X to M_1_ to Y_2_	**1.62 (1.23, 2.86)**	X to M_1_ to Y_2_	**1.48 (1.15, 2.34)**
Indirect Effect through Depressive/Anxious Symptoms	X to M_21_ to Y_2_	0.80 (0.55, 1.05)	X to M_22_ to Y_2_	0.96 (0.73, 1.19)
Indirect Effect through Depressive Symptoms and Depressive/Anxious Symptoms	X to M_1_ to M_21_ to Y_2_	0.92 (0.78, 1.01)	X to M_1_ to M_22_ to Y_2_	1.00 (0.95, 1.03)
	**Marijuana Use (Y**_**3**_ **)**			
	**Depressive Symptoms (M**_**21**_ **)**		**Anxious Symptoms (M**_**22**_ **)**	
**Measures**	**Path**	**OR (95% CI)**	**Path**	**OR (95% CI)**
Gender Minority Stressor Composite to Internalized Transphobia	a_11_ (X to M_1_)	**1.58 (1.34, 1.87)**	a_11_ (X to M_1_)	**1.58 (1.34, 1.87)**
Gender Minority Stressor Composite to Depressive/Anxious Symptoms	a_12_ (X to M_21_)	**1.55 (1.32, 1.82)**	a_12_ (X to M_22_)	**1.38 (1.13, 1.67)**
Internalized Transphobia to Marijuana Use	b_1_ (M_1_ to Y_3_)	**1.92 (1.11, 3.33)**	b_1_ (M_1_ to Y_3_)	**2.10 (1.24, 3.56)**
Depressive/Anxious Symptoms to Marijuana Use	b_2_ (M_21_ to Y_3_)	1.00 (0.56, 1.80)	b_2_ (M_22_ to Y_3_)	0.43 (0.22, 0.83)
Internalized Transphobia to Depressive/Anxious Symptoms	d (M_1_ to M_21_)	**1.41 (1.20, 1.66)**	d (M_1_ to M_22_)	1.07 (0.88, 1.30)
Gender Minority Stressor Composite to Marijuana Use	c’ (X to Y_3_)	0.82 (0.47, 1.44)	c’ (X to Y_3_)	1.11 (0.63, 1.97)
Indirect Effect through Internalized Transphobia	X to M_1_ to Y_3_	**1.35 (1.06, 1.88)**	X to M_1_ to Y_3_	**1.40 (1.13, 2.01)**
Indirect Effect through Depressive/Anxious Symptoms	X to M_21_ to Y_3_	1.00 (0.78, 1.34)	X to M_22_ to Y_3_	0.76 (0.49, 0.91)
Indirect Effect through Depressive Symptoms and Depressive/Anxious Symptoms	X to M_1_ to M_21_ to Y_3_	1.00 (0.91, 1.12)	X to M_1_ to M_22_ to Y_3_	0.97 (0.90, 1.05)

^1^Tobacco models controlled for sex assigned at birth; alcohol and marijuana models controlled for both wave and sex assigned at birth. Significant effects are bolded.
