# Supplementary material for: Longitudinal effects of gender minority stressors on substance use and related risk and protective factors among gender minority adolescents
Source: PLoS One. 2021 Jun 2;16(6):e0250500. doi: 10.1371/journal.pone.0250500 (PMC8171963; doi:10.1371/journal.pone.0250500)
Supplement: S1 Table — (DOCX) [file pone.0250500.s001.docx]

S1 Table

*Goodness-of-Fit (QIC) for Bivariate Associations Among Gender Minority Stressors and Hypothesized Risk and Protective Factors*

| Measures | 1 | 2 | 3 | 4 | 5 | 6 | 7 | 8 | 9 |
| --- | --- | --- | --- | --- | --- | --- | --- | --- | --- |
| 1. Gender minority stressor composite | 1 |  |  |  |  |  |  |  |  |
| 2. Internalized transphobia | 138.0 | 1 |  |  |  |  |  |  |  |
| 3. Depressive symptoms | 137.6 | 137.7 | 1 |  |  |  |  |  |  |
| 4. Anxious symptoms | 139.1 | 137.2 | 139.4 | 1 |  |  |  |  |  |
| 5. Resilience | 138.9 | 138.5 | 139.0 | 139.4 | 1 |  |  |  |  |
| 6. Gender-related pride | 138.5 | 141.8 | 139.0 | 138.3 | 139.5 | 1 |  |  |  |
| 7. Family functioning | 137.0 | 139.5 | 138.5 | 139.6 | 140.4 | 139.5 | 1 |  |  |
| 8. Social support | 139.4 | 138.1 | 138.3 | 139.0 | 138.7 | 139.2 | 141.1 | 1 |  |
| 9. Gender-related community connectedness | 138.2 | 136.9 | 138.3 | 138.9 | 138.5 | 138.9 | 139.3 | 138.6 | 1 |
